# Hertel Exophthalmometry Values in a Greek Adult Outpatient Clinic-Based Population: Association With Demographic Factors and Systemic Disease

**DOI:** 10.7759/cureus.35027

**Published:** 2023-02-15

**Authors:** Anastasia Tsiogka, Petros Petrou, Konstantinos Droutsas, Anthi Nikolopoulou, Dimitrios Papaconstantinou, Klio I Chatzistefanou

**Affiliations:** 1 Ophthalmology, 401 General Military Hospital of Athens, Athens, GRC; 2 Ophthalmology, General Hospital of Athens "G. Gennimatas", Athens, GRC; 3 Ophthalmology, National and Kapodistrian University of Athens, Athens, GRC; 4 Strabismus Service, General Hospital of Athens "G. Gennimatas", Athens, GRC; 5 Ophthalmology, Strabismus Service, General Hospital of Athens "G. Gennimatas", Athens, GRC

**Keywords:** orbital adipose tissue, thyroid dysfunction, arterial hypertension, diabetes mellitus, exophthalmometry

## Abstract

Purpose: To investigate correlations of exophthalmometry values (EVs) with age, gender, and the presence of diabetes mellitus, arterial hypertension, and dyslipidemia.

Methods: In a cross-sectional, clinic-based study, consecutive adult Greek patients presenting for evaluation at the outpatient general clinic on a scheduled appointment basis at a tertiary care referral center were submitted to Hertel exophthalmometry in both eyes by the same observer. Subjects with signs of history or orbital pathology, including thyroid-associated ophthalmopathy, were excluded. Demographics, as well as a detailed systemic history report, were recorded. Mixed effect linear regression analysis was performed to account for the correlation between the eyes of the same participant.

Results: A total of 800 eyes (400 subjects) were included, 194 males and 206 females, with a mean age of 67.82 ± 12 years (range: 18-92 years). The mean exophthalmometry value was 15.7 ± 2.6 mm (range: 11-21 mm). Every one year of increase in age is associated with a decrease in EVs by 0.03 mm (95% CI -0.04, -0.02/p-value<0.001). Female gender was associated with lower EVs by 0.33mm (95% CI-0.56, -0.1/p-value=0.005). Patients with diabetes mellitus had higher EVs by 0.47 mm (95% CI 0.25, 0.70/p-value<0.001) compared to patients without diabetes, and patients with arterial hypertension had lower EVs by 0.26 mm (95% CI -0.5, -0.02/p-value=0.034) compared to patients without hypertension. No association was found between dyslipidemia and systemic history of thyroid dysfunction.

Conclusions: A negative correlation of EVs was noted with increasing age, female gender, as well as history of arterial hypertension and a positive correlation with diabetes mellitus.

## Introduction

The orbit is a protective bony socket in the skull that contains and protects the eyeball, the optic nerve, other nerves of the eye, adipose tissue (fat), blood vessels, and six extraocular muscles [1,2]. It is pear-shaped, and its volume is 30cc [1]. Enlargement of one or more elements of the orbital tissue can cause ocular protrusion [1]. Exophthalmometry is a routine examination method for patients with suspected ocular protrusion [3]. The Hertel exophthalmometer is a reliable method for measuring ocular protrusion [4] and the most commonly used exophthalmometer in clinical settings [5]. Normal exophthalmometry values are trying to be established by the scientific community for different populations [4]. Normative exophthalmometry values (EVs) may vary and may be affected by ethnic origin, age, gender, refraction, and axial length of the eye [2,4]. Well-known pathological conditions causing proptosis include thyroid-associated orbitopathy, head and orbital trauma, tumors, and craniofacial abnormalities [2,6]. The purpose of this study was to determine exophthalmometry values in the Greek population and the impact of age, gender, and common systemic diseases, including diabetes mellitus, arterial hypertension, and dyslipidemia.

## Materials and methods

Study design and population

As a part of a larger ongoing study designed to characterize normative values for exophthalmometry readings in the Greek population, we randomly recruited patients presenting at the outpatient general clinic on a scheduled appointment basis at a tertiary care referral center (Athens General Hospital “G. Gennimatas”).

The study was approved by the Institutional Review Board of “G. Gennimatas” General Hospital of Athens and was conducted in accordance with the Declaration of Helsinki. Recruiting and assessment took place between January 2017 and December 2019. All subjects were informed and provided their consent before enrolling in the study.

Inclusion criteria were age greater than 18 years, Caucasian origin, and absence of medical or surgical retinal intervention for a time interval shorter than four weeks before the exophthalmometry measurement. Exclusion criteria were: history of strabismus or orbital pathology, including thyroid-associated ophthalmopathy, orbital tumors, orbital trauma, and craniofacial abnormalities or asymmetry, as well as EVs difference between the two eyes equal or greater than 2 mm.

Data collection and outcome measures

Demographic information (age, gender, race) and a detailed systemic history were obtained for all study participants. Patient history was obtained and recorded by recruiting physicians (KC, P.P) while the patients waited for evaluation. Participants were specifically asked about the presence of thyroid dysfunction and systemic vasculopathy disease, including arterial hypertension, diabetes mellitus, and dyslipidemia; evidence of the above was further supported by documentation of pertinent, concurrent, or past medical and/or past surgical treatment (i.e., thyroidectomy for thyroid dysfunction). The absence of thyroid-associated orbitopathy was based on the patient's reported history of absence of obvious ocular pathology, including exophthalmos and diplopia, as well as the absence of reported concurrent or past medical or surgical treatment suggestive of thyroid-associated orbitopathy, including orbit, extraocular muscles, or eyelid surgery, corticosteroid, immunosuppressive or selenium administration.

All subjects underwent Hertel exophthalmometry in both eyes (Hertel exophthalmometer K-0161, Inami& Co. Ltd. Japan). Exophthalmometry readings to the closest millimeter (mm) were measured at one setting for both eyes; the distance between the lateral canthi (intercanthal distance, ICD), as well as the interpupillary distance (IPD), were measured by the same observer (AN) and recorded as well.

Statistical analysis

The normal distribution of demographic and clinical information was assessed by plots (histogram and probability graphs) and corresponding statistical tests (Kolmogorov-Smirnov/Shapiro-Wilk test). Normally distributed continuous values were summarized by the mean and standard deviation (SD), and categorical data by number (N) and presentence (%). The correlation between EVs and all studied variables was investigated by applying univariate and multivariate linear regression analysis for each eye separately and for both eyes together. To assess the sensitivity of our findings for the latter analysis, we further applied mixed effects linear regression to account for the correlation between the eyes from the same subject. All multivariate models were adjusted for age, gender, diabetes mellitus, arterial hypertension, thyroid dysfunction, and dyslipidemia. Statistical significance was set at P<0.05. Analysis was conducted in StataCorp. 2013. Stata Statistical Software: Release 13. College Station, TX: StataCorp LP.

## Results

From 430 subjects who initially consented, 409 were eligible to participate in the study. Patients who demonstrated poor cooperation with exophthalmometry testing, some of them with unstable fixation due to low vision, four subjects with Asian racial backgrounds, and five subjects with an exophthalmometry value difference of equal or larger than 2 mm between the two eyes were excluded. Hence a total of 400 patients (800 eyes) were enrolled in the study. All patients were of Greek (Caucasian) ethnicity.

The majority of patients were under medical treatment for the underlying medical condition: 182 (45.50%) were diagnosed with diabetes mellitus, 173 (43.25%) with arterial hypertension, 121 (30.25%) with dyslipidemia and 57 (14.25%) with thyroid dysfunction. The clinical characteristics of the studied variables under investigation are demonstrated in Table 1.

**Table 1:** Clinical characteristics of the studied variables

		N (%)	Exophthalmometry values (Mean±SD)
Gender	Males	194 (48.50)	16.07±1.67
	Females	206 (51.50)	15.60±1.66
Diabetes Mellitus	No	218 (54.50)	15.59±1.63
	Yes	182 (45.50)	16.10± 1.70
Arterial Hypertension	No	227 (56.75)	16±1.68
	Yes	173 (43.25)	15.61±1.66
Thyroid dysfunction	No	343 (85.75)	15.87±1.68
	Yes	57 (14.25)	15.54±1.67
Dyslipidemia	No	279 (69.75)	15.84±1.69
	Yes	121 (30.25)	15.80±1.66
N=Number; SD=standard deviation			

The mean Hertel exophthalmometry value was 15.8±1.7 mm (range; 12 to 22 mm). The mean interpupillary distance was 60.97±2.5 (range; 55-70), and the mean intercanthal distance was 97.81 ± 3.82 mm (range; 90 to 112 mm). The mean difference of the exophthalmometry value (relative exophthalmometric value) between the eyes of each participant was 0.08 ± 0.06 mm in the study population, with a maximum difference of 1.5 mm.

**Figure 1:**
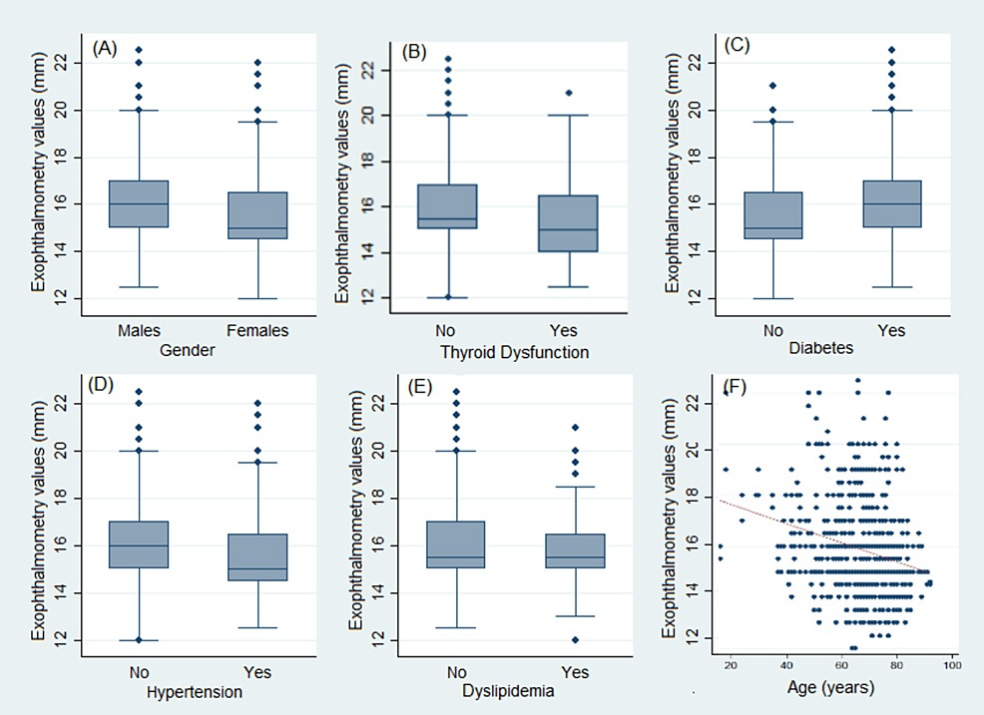
Boxplots of absolute exophthalmometry values of all eyes involved (N=800) with respect to gender (A), thyroid dysfunction (B), diabetes (C), hypertension, (D) and dyslipidemia (E) and a scatterplot of absolute exophthalmometry values of all eyes involved (N=800) in respect to age (F).

We performed univariate linear regression analysis between EVs and each of the variables studied (only for the left eyes, only for the right eyes, and for both eyes, respectively). There was a statistically significant negative correlation between EVs and age, female gender, and arterial hypertension and a statistically significant positive correlation with diabetes mellitus in each analysis. In the univariate analysis for both eyes, thyroid dysfunction had a bordered statistically significant negative correlation with EVs. Univariate analysis of the left and right eye separately for thyroid dysfunction and each univariate analysis for dyslipidemia were not proven of statistical significance. The results are presented in Table 2. Scatterplots of exophthalmometric values with respect to age and box plots of the descriptive relationship between EVs and thyroid dysfunction, diabetes, hypertension, and dyslipidemia are presented in Figure 1.

**Table 2:** Univariate linear regression analysis between exophthalmometry values and each of the studied variables (only for left eyes, only for right eyes, and for both eyes, respectively)

Exophthalmometry values	Only left			Only right			Both eyes		
	Coef	p-value	95%CI	Coef	p-value	95%CI	Coef	p-value	95%CI
Age	-0.04	<0.001***	-0.05, -0.02	-0.04	<0.001***	-0.05, -0.02	-0.04	<0.001***	-0.04, -0.03
Gender (females)	-0.48	0.004**	-0.8, -0.16	-0.5	0.006**	-0.8, -0.13	-0.5	<0.001***	-0.7, -0.24
Diabetes	0.48	0.004**	0.2, 0.8	0.55	0.001**	0.22, 0.88	0.5	<0.001***	0.28, 0.74
Hypertension	-0.4	0.017*	-0.7, -0.07	-0.36	0.032*	-0.7, -0.03	-0.4	0.001**	-0.62, -0.15
Dyslipidemia	-0.01	0.940	-0.4, 0.35	-0.06	0.747	-0.42, 0.3	-0.04	0.778	-0.29, 0.22
Thyroid Dysfunction	-0.45	0.063	-0.92, 0.02	-0.23	0.340	-0.7, 0.24	-0.34	0.047*	-0.67, -0.005
***p<0.001, **p<0.01, *p<0.05; Coef= coefficient; CI=confidence intervals									

We also performed multivariable linear regression analysis among EVs; and all the variables studied (only left eyes, only right eyes, or both eyes, respectively). The above-noted correlations of univariate linear regression analysis of EVs with age, presence of diabetes mellitus, and arterial hypertension persisted. The bordered correlation with thyroid dysfunction no longer existed in the multivariate linear regression analysis.

Finally, we applied mixed-effects linear regression adjusted for each variable to account for the dependence that both eyes correspond to the same individual (Table 3). Analysis revealed that the mixed effects linear regression model was the optimal fit that better accounted for the structure of the observation since the likelihood ratio test versus linear regression showed a statistically significant difference (p-value<0.001) in every comparison, keeping all other confounders not statistically significant.

**Table 3:** Mixed effects linear regression model adjusted for each studied variable

Exophthalmometry values			
	Coef.	p-value	95% CI
Age	-0.03	<0.001***	-0.04, -0.02
Gender (females)	-0.33	0.005 **	-0.56, -0.1
Diabetes	0.47	<0.001***	0.25, 0.70
Hypertension	-0.26	0.030*	-0.5, -0.02
Dyslipidemia	0.11	0.395	-0.14, 0.36
Thyroid Dysfunction	-0.23	0.172	-0.55, 0.1
***p<0.001, **p<0.01, *p<0.05; Coef= coefficient; CI=confidence intervals			

We conclude that there was a statistically significant negative correlation between EVs and age, female gender, and arterial hypertension and a statistically significant positive correlation with diabetes mellitus. More specifically, every one year of increase in age is associated with a decrease in exophthalmometry measurements by 0.03 mm (95% CI -0.04, -0.02/ p-value<0.001). Females had decreased EVs by 0.33mm (95% CI-0.56, -0.1 / p-value=0.005). Moreover, patients with hypertension had lower EVs by 0.26 mm (95% CI -0.5, -0.02/ p-value=0.030 compared to patients without hypertension. In addition, patients with diabetes had a higher exophthalmometry measurement by 0.47 mm (95% CI 0.25, 0.70/ p-value<0.001) compared to patients without diabetes. Thyroid dysfunction and dyslipidemia were not proven of statistical significance.

We also performed multivariate and mixed-effect linear regression analysis adding intercanthal and interpupillary distance to the above-noted variables. The above-noted correlations of EVs with age, presence of diabetes mellitus, and arterial hypertension persisted the negative correlation with female gender no longer persisted in the multivariate and mixed-effects regression analysis. This was the result of the preponderance of the correlation of the variable “intercanthal distance” as a statistically significant positive correlation with EVs.

## Discussion

In this clinic-based cross-sectional study, common vasculopathy systemic diseases were found to be correlated with differences in exophthalmometry measurements. More specifically, arterial hypertension was negatively correlated with EVs, while diabetes mellitus was positively correlated. To our knowledge, this is the first study that evaluates exophthalmometry in relation to diabetes mellitus, arterial hypertension, and dyslipidemia.

The relationship of exophthalmometric values with demographic factors has been extensively investigated. Kashkouli et al. [7] and Nath et al. [8] demonstrated that children and teenagers EVs significantly increased as they grow older into adulthood. Fledelius [9] found EVs to remain stable after the late teenage years. Several studies have demonstrated a negative correlation between EVs and advancing age, reporting a statistically significant reduction in exophthalmolmetry values with every year of age increase starting from 20 years of age [4,10-12]. Our study results are consistent with these findings.

The association between gender and ocular protrusion remains controversial. Some studies [4,13-15] reported significantly higher values in males than in females. Kashkouli et al. [3] and Wu et al. [12] did not find a statistically significant correlation between gender and EVs in their study. Our study demonstrated statistically significantly lower EVs in female participants, a finding related to facial metric parameters since it was overshadowed by statistically significant positive associations of exophthalmometric values with intercanthal distance measurements.

Ethnic and racial background may affect facial metric parameters [13,16]; we thus opted for excluding the few number subjects of non-Caucasian origin who had initially been recruited in this study.

In a report on 46 participants by Detorakis et al. [17], the mean exophthalmometry value was reported to be 16 mm for males and 15mm for females, in a hospital-based sample of the Greek adult population, with differences between gender not being statistically significant. In addition, the EVs detected in the current study were comparable to those described by other studies. In a cross-sectional study on 236 Turkish adult participants by Karti et al. [2], mean Hertel exophthalmometric readings were 15.7±2.6 mm (range; 11 to 21 mm). The mean value for males was 16.1±2.6 mm, and for females, 15.5±2.6 mm. Bageri et al. [10], in a metanalysis of four studies in the Iranian population and 3,696 subjects, reported a mean exophthalmometry value for males at 16.5 mm and females at 16.2 mm. Barrette et al. [17], in a cohort that included 65 white adults, reported mean EVs of 17±2.65 mm for white males and 15.98±2.22 mm for white females.

Other than thyroid eye disease, certain systemic diseases have been reported to affect eye protrusion. Schwarz et al. [18] postulated that there was possibly an exophthalmos-producing activity in the serum and pituitary of patients with Cushing's syndrome and acromegaly. Obesity has been shown to enhance ocular protrusion [15,19]. Smolders et al. [19] demonstrated that 33% of obese patients in their series had bilateral exophthalmos.

Obesity is a predisposing factor for diabetes mellitus and arterial hypertension [20]. Type 2 diabetes mellitus is an expanding global health problem. The most common ophthalmic complications of longstanding diabetes mellitus, macular edema, and proliferative diabetic retinopathy are associated with the thickening of intraocular structures, including the retina [21]; reports on the impact of diabetes on the choroid are controversial [21-23].

The extensive investigation of diabetic complications on the thickness of intraocular structures has led investigators to propose specific choroidal imaging indices as biomarker candidates. Endo et al. [23], using enhanced depth imaging optical coherence tomography and a binarisation method, suggested a potential biomarker role for the ratio of the luminal area (LA) in the total choroidal area (TCA), designated as the L/C ratio: they found that a lower L/C ratio was associated with longer duration of diabetes.

On the other hand, there is no data in access to our literature on the impact of diabetes mellitus on the orbital soft tissues. A majority of individuals suffering from type 2 diabetes are obese, with central visceral adiposity [24]. Several adipose-tissue-centric mechanisms have been proposed in the pathogenesis of diabetes mellitus, with chronic, low-grade adipose tissue inflammation receiving considerable attention [25]. Although the intraorbital fat has not been the subject of related investigations [26], being assigned to a different origin, the neural crest as opposed to mesoderm for the most well-studied subcutaneous or visceral adipose tissue [27], we might hypothesize that orbital adipose perivascular tissue could potentially be involved in low-grade chronic inflammation in diabetic patients accounting for the relative increase in noted EVs series.

It is also noteworthy that the presence of diabetes mellitus has been associated with more severe forms of thyroid-associated ophthalmopathy [28]. Side effects of diabetic medications could also be considered as possible contributors to ocular proptosis. Dorkhan et al. [29] described a subgroup of type 2 diabetic patients responding with increased eye protrusion when treated with pioglitazone, one of the thiazolidinediones, a class of glucose-lowering drugs that promote insulin sensitivity.

Arterial hypertension was shown to correlate with lower EVs in our study. There is extensive literature associating elevated blood pressure with decreased thickness in intraocular structures, including decreased choroidal thickness [30], decreased macular thickness [31], inner retinal layer, particularly ganglion cell-inner plexiform layer (GC-IPL) thinning [32], which is significantly correlated with a decrease in retinal blood flow [33]. Antihypertensive medication, specifically angiotensin-converting enzyme inhibitors or diuretics, was reported to be associated with thinning of the retinal nerve fiber layer [32]. The above well-studied and reproduced associations have led to suggesting a possible role of Optical Coherence Tomography angiography in identifying subclinical microvascular damage [34].

On the antipodes of extensive investigation regarding intraocular structures, there is a paucity of data on the impact of arterial hypertension on extraocular orbital tissues in the access to our literature. We could theorize that arteriosclerosis-related reduction in vascular flow in the intraorbital soft tissues associated with systemic arterial hypertension could account for decreased EVs in our series.

It is noteworthy that the presence of isolated systemic thyroid dysfunction (without signs of thyroid-associated orbitopathy) was not associated with a change in exophthalmometry readings either in the multivariate or the mixed-effects regression analysis in this study. We postulate that these data indirectly support the statistical significance of our findings on the associations with arterial hypertension and diabetes mellitus. Dyslipidemia was not found to be correlated with the exophthalmometry readings either.

The knowledge that certain systemic diseases (diabetes mellitus and arterial hypertension) but not others (dyslipidemia or thyroid dysfunction without signs of thyroid-associated orbitopathy) may affect exophthalmometry readings may be of value in the interpretation of exophthalmometry when coexisting orbital or systemic (dysthyroid) pathology is contemplated. The above-noted associations may also shed light on a better understanding of the pathophysiology of diabetes mellitus and the impact of microvascular systemic disease in the microenvironment of the orbit.

This study has to be viewed in light of its limitations. Systemic history was based on a patient-reported history. Several possible confounding factors, including obesity, were not documented in this study. The exact concomitant retinal disease diagnosis, as well as a detailed medication intake history, were not recorded and analyzed. Medication history is important because apart from systemic, topical ophthalmic medications have been associated with a change in ocular protrusion as well: use of prostaglandin intraocular pressure lowering drops, for example, notably bimatoprost, have been shown to decrease orbital fat and induce enophthalmos [35].

The external validity of our results needs to be confirmed, as measurements have been taken on a selected patient sample. Possible bias towards more severe and longstanding cases of diabetes mellitus or hypertension, as evidenced by their complicated course and referral for ophthalmic manifestations, may exist.

Future studies could validate our results by expanding to a population-based sample and addressing additional confounding factors, including specific ophthalmic diagnosis, the axial length of the eye, body mass indices, and a detailed systemic and topical medication intake history.

## Conclusions

In this clinic-based cross-sectional study, a negative correlation of EVs was noted with increasing age, female gender, as well a history of arterial hypertension, and a positive correlation with diabetes mellitus. To our knowledge, this is the first study that evaluates exophthalmometry in relation to common vasculopathy systemic diseases. Future studies could validate our results by expanding to a population-based sample and addressing additional confounding factors.
